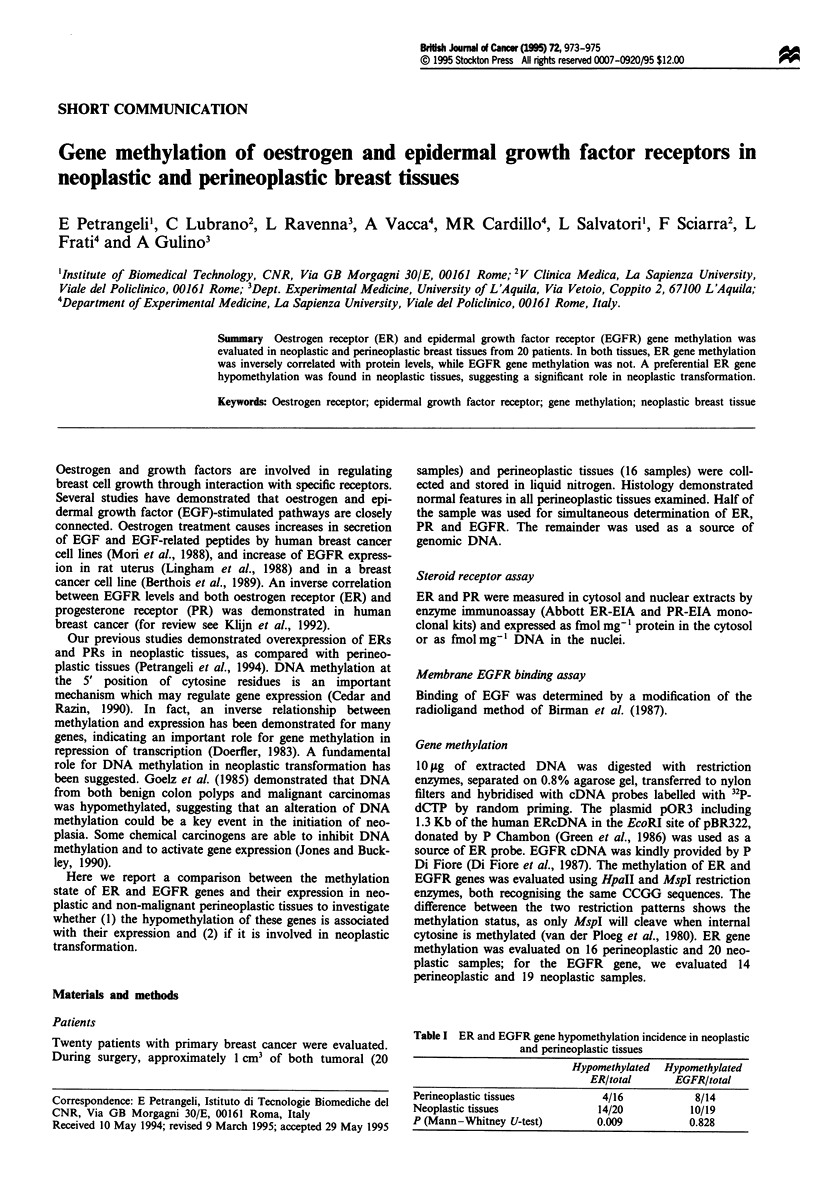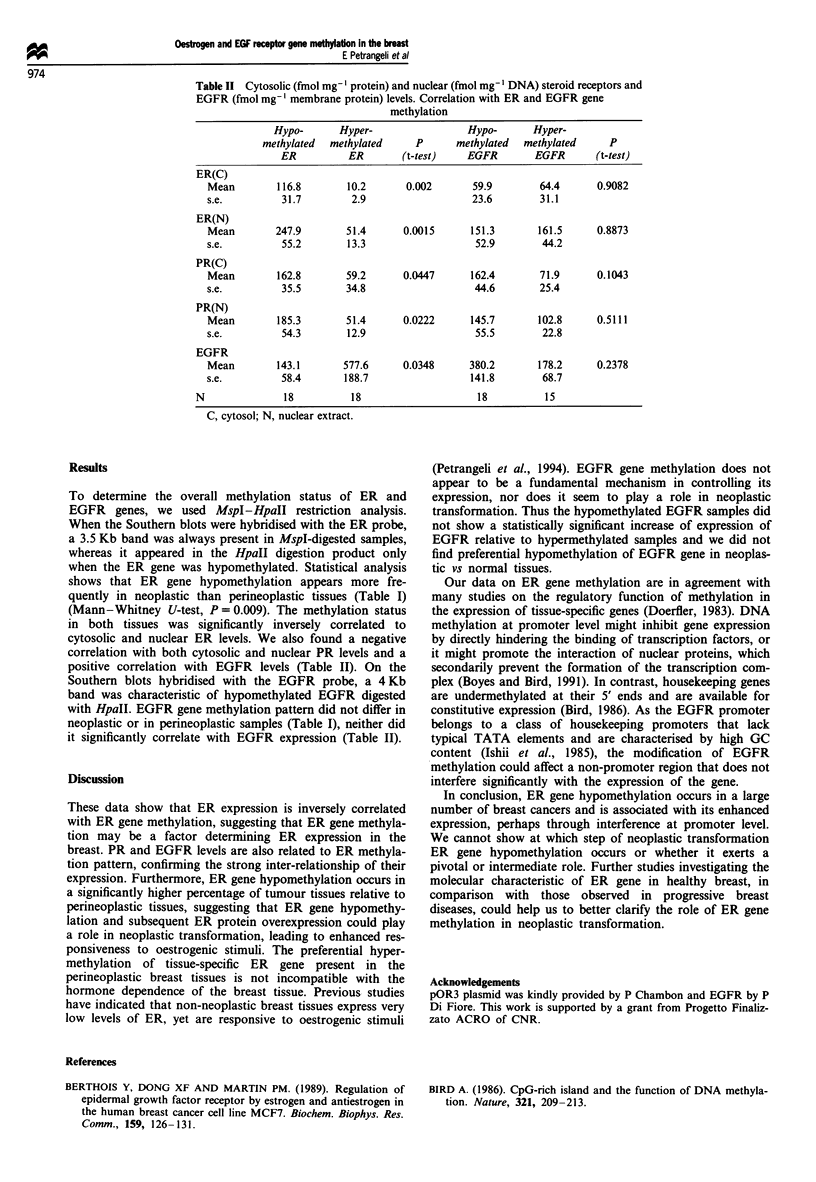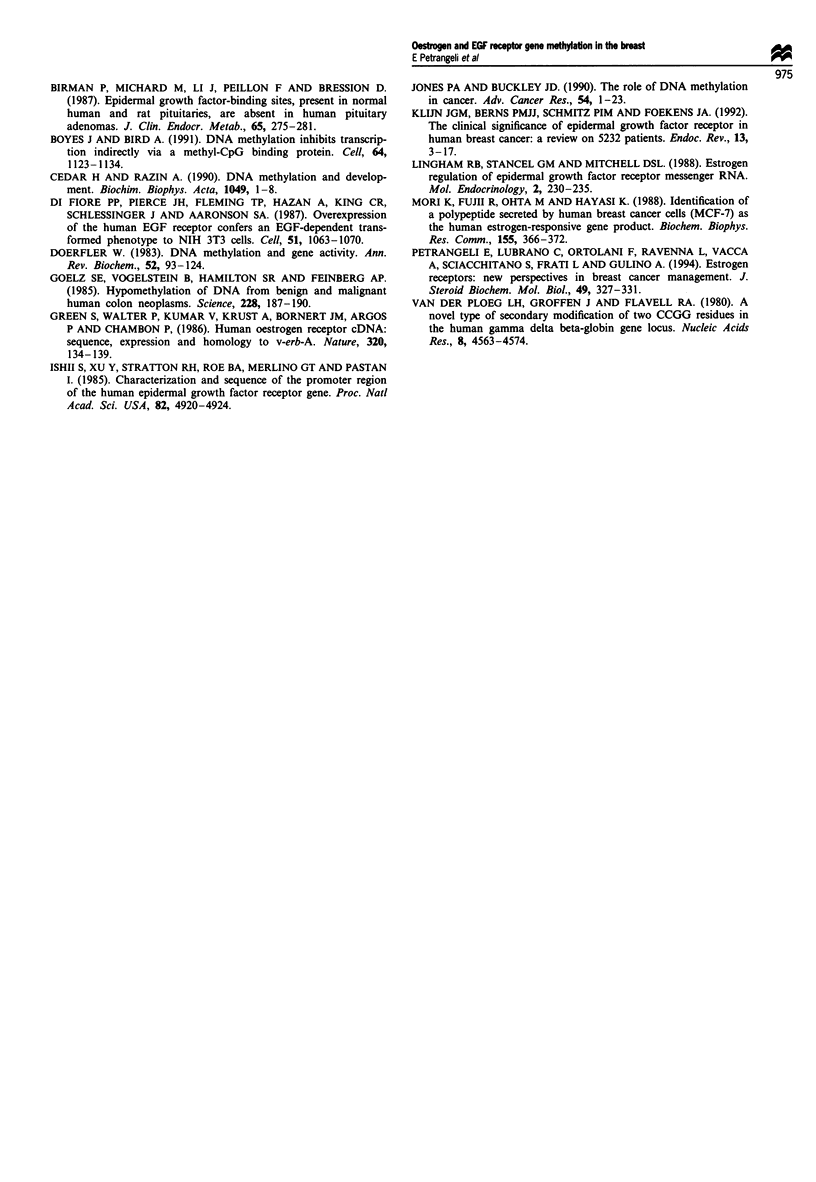# Gene methylation of oestrogen and epidermal growth factor receptors in neoplastic and perineoplastic breast tissues.

**DOI:** 10.1038/bjc.1995.444

**Published:** 1995-10

**Authors:** E. Petrangeli, C. Lubrano, L. Ravenna, A. Vacca, M. R. Cardillo, L. Salvatori, F. Sciarra, L. Frati, A. Gulino

**Affiliations:** Institute of Biomedical Technology, CNR, Rome, Italy.

## Abstract

Oestrogen receptor (ER) and epidermal growth factor receptor (EGFR) gene methylation was evaluated in neoplastic and perineoplastic breast tissues from 20 patients. In both tissues, ER gene methylation was inversely correlated with protein levels, while EGFR gene methylation was not. A preferential ER gene hypomethylation was found in neoplastic tissues, suggesting a significant role in neoplastic transformation.


British Journal of Cancer (1995) 72, 973-975
© 1995 Stockton Press All rights reserved 0007-0920/95 $12.00

SHORT COMMUNICATION

### Gene methylation of oestrogen and epidermal growth factor receptors in neoplastic and perineoplastic breast tissues

E Petrangeli[1], C Lubrano[2], L Ravenna[3], A Vacca[4], MR Cardillo[4], L Salvatori[1], F Sciarra[2], L Frati[4] and A Gulino[3]

[1]*Institute of Biomedical Technology, CNR, Via GB Morgagni 30/E, 00161 Rome;* [2]*V Clinica Medica, La Sapienza University, Viale del Policlinico, 00161 Rome;* [3]*Dept. Experimental Medicine, University of L'Aquila, Via Vetoio, Coppito 2, 67100 L'Aquila;* [4]*Department of Experimental Medicine, La Sapienza University, Viale del Policlinico, 00161 Rome, Italy.*

**Summary** Oestrogen receptor (ER) and epidermal growth factor receptor (EGFR) gene methylation was evaluated in neoplastic and perineoplastic breast tissues from 20 patients. In both tissues, ER gene methylation was inversely correlated with protein levels, while EGFR gene methylation was not. A preferential ER gene hypomethylation was found in neoplastic tissues, suggesting a significant role in neoplastic transformation.

**Keywords:** Oestrogen receptor; epidermal growth factor receptor; gene methylation; neoplastic breast tissue

Oestrogen and growth factors are involved in regulating breast cell growth through interaction with specific receptors. Several studies have demonstrated that oestrogen and epidermal growth factor (EGF)-stimulated pathways are closely connected. Oestrogen treatment causes increases in secretion of EGF and EGF-related peptides by human breast cancer cell lines (Mori *et al.*, 1988), and increase of EGFR expression in rat uterus (Lingham *et al.*, 1988) and in a breast cancer cell line (Berthois *et al.*, 1989). An inverse correlation between EGFR levels and both oestrogen receptor (ER) and progesterone receptor (PR) was demonstrated in human breast cancer (for review see Klijn *et al.*, 1992).

Our previous studies demonstrated overexpression of ERs and PRs in neoplastic tissues, as compared with perineoplastic tissues (Petrangeli *et al.*, 1994). DNA methylation at the 5′ position of cytosine residues is an important mechanism which may regulate gene expression (Cedar and Razin, 1990). In fact, an inverse relationship between methylation and expression has been demonstrated for many genes, indicating an important role for gene methylation in repression of transcription (Doerfler, 1983). A fundamental role for DNA methylation in neoplastic transformation has been suggested. Goelz *et al.* (1985) demonstrated that DNA from both benign colon polyps and malignant carcinomas was hypomethylated, suggesting that an alteration of DNA methylation could be a key event in the initiation of neoplasia. Some chemical carcinogens are able to inhibit DNA methylation and to activate gene expression (Jones and Buckley, 1990).

Here we report a comparison between the methylation state of ER and EGFR genes and their expression in neoplastic and non-malignant perineoplastic tissues to investigate whether (1) the hypomethylation of these genes is associated with their expression and (2) if it is involved in neoplastic transformation.

### Materials and methods

### *Patients*

Twenty patients with primary breast cancer were evaluated. During surgery, approximately 1 $cm^3$ of both tumoral (20 samples) and perineoplastic tissues (16 samples) were collected and stored in liquid nitrogen. Histology demonstrated normal features in all perineoplastic tissues examined. Half of the sample was used for simultaneous determination of ER, PR and EGFR. The remainder was used as a source of genomic DNA.

### *Steroid receptor assay*

ER and PR were measured in cytosol and nuclear extracts by enzyme immunoassay (Abbott ER-EIA and PR-EIA monoclonal kits) and expressed as fmol $mg^{-1}$ protein in the cytosol or as fmol $mg^{-1}$ DNA in the nuclei.

### *Membrane EGFR binding assay*

Binding of EGF was determined by a modification of the radioligand method of Birman *et al.* (1987).

### *Gene methylation*

10 μg of extracted DNA was digested with restriction enzymes, separated on 0.8% agarose gel, transferred to nylon filters and hybridised with cDNA probes labelled with $^{32}P$-dCTP by random priming. The plasmid pOR3 including 1.3 Kb of the human ERcDNA in the *Eco*RI site of pBR322, donated by P Chambon (Green *et al.*, 1986) was used as a source of ER probe. EGFR cDNA was kindly provided by P Di Fiore (Di Fiore *et al.*, 1987). The methylation of ER and EGFR genes was evaluated using *Hpa*II and *Msp*I restriction enzymes, both recognising the same CCGG sequences. The difference between the two restriction patterns shows the methylation status, as only *Msp*I will cleave when internal cytosine is methylated (van der Ploeg *et al.*, 1980). ER gene methylation was evaluated on 16 perineoplastic and 20 neoplastic samples; for the EGFR gene, we evaluated 14 perineoplastic and 19 neoplastic samples.

**Table I** ER and EGFR gene hypomethylation incidence in neoplastic and perineoplastic tissues

| | *Hypomethylated ER/total* | *Hypomethylated EGFR/total* |
|---|---|---|
| Perineoplastic tissues | 4/16 | 8/14 |
| Neoplastic tissues | 14/20 | 10/19 |
| *P* (Mann–Whitney *U*-test) | 0.009 | 0.828 |

Correspondence: E Petrangeli, Istituto di Tecnologie Biomediche del CNR, Via GB Morgagni 30/E, 00161 Roma, Italy
Received 10 May 1994; revised 9 March 1995; accepted 29 May 1995

**Table II** Cytosolic (fmol mg$^{-1}$ protein) and nuclear (fmol mg$^{-1}$ DNA) steroid receptors and EGFR (fmol mg$^{-1}$ membrane protein) levels. Correlation with ER and EGFR gene methylation

| | *Hypo-methylated ER* | *Hyper-methylated ER* | *P (t-test)* | *Hypo-methylated EGFR* | *Hyper-methylated EGFR* | *P (t-test)* |
|---|---|---|---|---|---|---|
| ER(C) | | | | | | |
| Mean | 116.8 | 10.2 | 0.002 | 59.9 | 64.4 | 0.9082 |
| s.e. | 31.7 | 2.9 | | 23.6 | 31.1 | |
| ER(N) | | | | | | |
| Mean | 247.9 | 51.4 | 0.0015 | 151.3 | 161.5 | 0.8873 |
| s.e. | 55.2 | 13.3 | | 52.9 | 44.2 | |
| PR(C) | | | | | | |
| Mean | 162.8 | 59.2 | 0.0447 | 162.4 | 71.9 | 0.1043 |
| s.e. | 35.5 | 34.8 | | 44.6 | 25.4 | |
| PR(N) | | | | | | |
| Mean | 185.3 | 51.4 | 0.0222 | 145.7 | 102.8 | 0.5111 |
| s.e. | 54.3 | 12.9 | | 55.5 | 22.8 | |
| EGFR | | | | | | |
| Mean | 143.1 | 577.6 | 0.0348 | 380.2 | 178.2 | 0.2378 |
| s.e. | 58.4 | 188.7 | | 141.8 | 68.7 | |
| N | 18 | 18 | | 18 | 15 | |

C, cytosol; N, nuclear extract.

### Results

To determine the overall methylation status of ER and EGFR genes, we used *Msp*I–*Hpa*II restriction analysis. When the Southern blots were hybridised with the ER probe, a 3.5 Kb band was always present in *Msp*I-digested samples, whereas it appeared in the *Hpa*II digestion product only when the ER gene was hypomethylated. Statistical analysis shows that ER gene hypomethylation appears more frequently in neoplastic than perineoplastic tissues (Table I) (Mann–Whitney $U$-test, $P = 0.009$). The methylation status in both tissues was significantly inversely correlated to cytosolic and nuclear ER levels. We also found a negative correlation with both cytosolic and nuclear PR levels and a positive correlation with EGFR levels (Table II). On the Southern blots hybridised with the EGFR probe, a 4 Kb band was characteristic of hypomethylated EGFR digested with *Hpa*II. EGFR gene methylation pattern did not differ in neoplastic or in perineoplastic samples (Table I), neither did it significantly correlate with EGFR expression (Table II).

### Discussion

These data show that ER expression is inversely correlated with ER gene methylation, suggesting that ER gene methylation may be a factor determining ER expression in the breast. PR and EGFR levels are also related to ER methylation pattern, confirming the strong inter-relationship of their expression. Furthermore, ER gene hypomethylation occurs in a significantly higher percentage of tumour tissues relative to perineoplastic tissues, suggesting that ER gene hypomethylation and subsequent ER protein overexpression could play a role in neoplastic transformation, leading to enhanced responsiveness to oestrogenic stimuli. The preferential hypermethylation of tissue-specific ER gene present in the perineoplastic breast tissues is not incompatible with the hormone dependence of the breast tissue. Previous studies have indicated that non-neoplastic breast tissues express very low levels of ER, yet are responsive to oestrogenic stimuli (Petrangeli *et al.*, 1994). EGFR gene methylation does not appear to be a fundamental mechanism in controlling its expression, nor does it seem to play a role in neoplastic transformation. Thus the hypomethylated EGFR samples did not show a statistically significant increase of expression of EGFR relative to hypermethylated samples and we did not find preferential hypomethylation of EGFR gene in neoplastic *vs* normal tissues.

Our data on ER gene methylation are in agreement with many studies on the regulatory function of methylation in the expression of tissue-specific genes (Doerfler, 1983). DNA methylation at promoter level might inhibit gene expression by directly hindering the binding of transcription factors, or it might promote the interaction of nuclear proteins, which secondarily prevent the formation of the transcription complex (Boyes and Bird, 1991). In contrast, housekeeping genes are undermethylated at their 5′ ends and are available for constitutive expression (Bird, 1986). As the EGFR promoter belongs to a class of housekeeping promoters that lack typical TATA elements and are characterised by high GC content (Ishii *et al.*, 1985), the modification of EGFR methylation could affect a non-promoter region that does not interfere significantly with the expression of the gene.

In conclusion, ER gene hypomethylation occurs in a large number of breast cancers and is associated with its enhanced expression, perhaps through interference at promoter level. We cannot show at which step of neoplastic transformation ER gene hypomethylation occurs or whether it exerts a pivotal or intermediate role. Further studies investigating the molecular characteristic of ER gene in healthy breast, in comparison with those observed in progressive breast diseases, could help us to better clarify the role of ER gene methylation in neoplastic transformation.

**Acknowledgements**
pOR3 plasmid was kindly provided by P Chambon and EGFR by P Di Fiore. This work is supported by a grant from Progetto Finalizzato ACRO of CNR.

### References

BERTHOIS Y, DONG XF AND MARTIN PM. (1989). Regulation of epidermal growth factor receptor by estrogen and antiestrogen in the human breast cancer cell line MCF7. *Biochem. Biophys. Res. Comm.*, **159**, 126–131.

BIRD A. (1986). CpG-rich island and the function of DNA methylation. *Nature*, **321**, 209–213.

BIRMAN P, MICHARD M, LI J, PEILLON F AND BRESSION D. (1987). Epidermal growth factor-binding sites, present in normal human and rat pituitaries, are absent in human pituitary adenomas. *J. Clin. Endocr. Metab.*, **65**, 275–281.

BOYES J AND BIRD A. (1991). DNA methylation inhibits transcription indirectly via a methyl-CpG binding protein. *Cell*, **64**, 1123–1134.

CEDAR H AND RAZIN A. (1990). DNA methylation and development. *Biochim. Biophys. Acta*, **1049**, 1–8.

DI FIORE PP, PIERCE JH, FLEMING TP, HAZAN A, KING CR, SCHLESSINGER J AND AARONSON SA. (1987). Overexpression of the human EGF receptor confers an EGF-dependent transformed phenotype to NIH 3T3 cells. *Cell*, **51**, 1063–1070.

DOERFLER W. (1983). DNA methylation and gene activity. *Ann. Rev. Biochem.*, **52**, 93–124.

GOELZ SE, VOGELSTEIN B, HAMILTON SR AND FEINBERG AP. (1985). Hypomethylation of DNA from benign and malignant human colon neoplasms. *Science*, **228**, 187–190.

GREEN S, WALTER P, KUMAR V, KRUST A, BORNERT JM, ARGOS P AND CHAMBON P, (1986). Human oestrogen receptor cDNA: sequence, expression and homology to v-*erb*-A. *Nature*, **320**, 134–139.

ISHII S, XU Y, STRATTON RH, ROE BA, MERLINO GT AND PASTAN I. (1985). Characterization and sequence of the promoter region of the human epidermal growth factor receptor gene. *Proc. Natl Acad. Sci. USA*, **82**, 4920–4924.

JONES PA AND BUCKLEY JD. (1990). The role of DNA methylation in cancer. *Adv. Cancer Res.*, **54**, 1–23.

KLIJN JGM, BERNS PMJJ, SCHMITZ PIM AND FOEKENS JA. (1992). The clinical significance of epidermal growth factor receptor in human breast cancer: a review on 5232 patients. *Endoc. Rev.*, **13**, 3–17.

LINGHAM RB, STANCEL GM AND MITCHELL DSL. (1988). Estrogen regulation of epidermal growth factor receptor messenger RNA. *Mol. Endocrinology*, **2**, 230–235.

MORI K, FUJII R, OHTA M AND HAYASI K. (1988). Identification of a polypeptide secreted by human breast cancer cells (MCF-7) as the human estrogen-responsive gene product. *Biochem. Biophys. Res. Comm.*, **155**, 366–372.

PETRANGELI E, LUBRANO C, ORTOLANI F, RAVENNA L, VACCA A, SCIACCHITANO S, FRATI L AND GULINO A. (1994). Estrogen receptors: new perspectives in breast cancer management. *J. Steroid Biochem. Mol. Biol.*, **49**, 327–331.

VAN DER PLOEG LH, GROFFEN J AND FLAVELL RA. (1980). A novel type of secondary modification of two CCGG residues in the human gamma delta beta-globin gene locus. *Nucleic Acids Res.*, **8**, 4563–4574.